# 2228. Knowledge, Attitudes, and Practices Regarding Antibiotic Use during the COVID-19 Pandemic: A Comparison of United States (US) Survey Respondents Living in the US-Mexico Border Region with those Living in Non-border Regions

**DOI:** 10.1093/ofid/ofad500.1850

**Published:** 2023-11-27

**Authors:** Maja Subelj, Jose Luis Camarena, Brooke A Hawkes, Katherine Ellingson

**Affiliations:** University of Ljubljana, Ljubljana, Ljubljana, Slovenia; University of Arizona College of Public Health, Tucson, Arizona; University of Arizona College of Public Health, Tucson, Arizona; University of Arizona, Tucson, Arizona

## Abstract

**Background:**

Inappropriate use of antibiotics is a driver of antimicrobial resistance globally. The US-Mexico border region is medically underserved and socially vulnerable. This study aimed to assess knowledge, attitudes and practices (KAP) regarding antibiotic use during the COVID-19 pandemic among US residents, and to compare KAPs for those living in border versus non-border regions.

**Methods:**

A cross-sectional online survey with monetary incentive was deployed to a sample of adults ≥18 years old through Amazon Mechanical Turk with enriched sampling in the US-Mexico border region (defined as residency within 100 km of the border). Surveys were completed from 8/8/2020 to 8/4/2021. KAP questions were aligned with published tools and COVID-19-specific questions were added. Of 602 respondents from the US, 590 (98%) had complete KAP data and were included in this analysis. Chi-square tests were performed to examine the association between KAPs related to antibiotic use and border residency status.

**Results:**

Overall, for 6 of 8 knowledge items about antibiotic use, fewer than 50% of survey respondents answered correctly (Table 1). 50% of border residents versus 71% of non-border residents (p< 0.01) correctly agreed that “antibiotics should never be saved for the next time you get sick.” Conversely 44% of border residents, versus 31% of non-border residents (p=0.03), correctly disagreed with the statement “when I have a cold, I should take antibiotics to avoid getting a more serious illness.” There was no difference in knowledge about causes of antibiotic resistance by region of residency. Overall, 40% of respondents believed that their personal antibiotic use did not affect antibiotic resistance (Table 2). Finally 37% all respondents said they tried to obtain antibiotics, and 31% tried to obtain chloroquine or hydroxychloroquine, because of concerns about COVID-19 with no difference by region of residency.

**Table 1:**
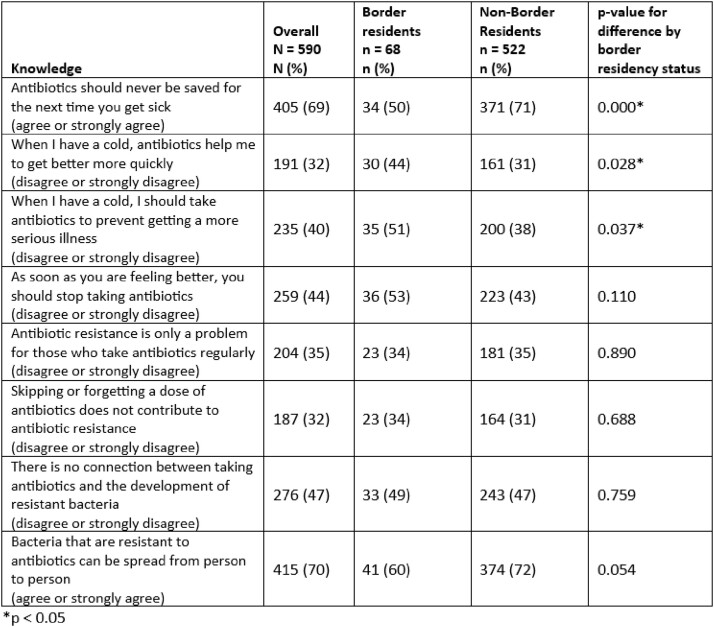
Number and percentage of survey respondents answering correctly to knowledge questions regarding antibiotic use, stratified by residency within 100 kilometers of the US-Mexico border.

**Table 2:**
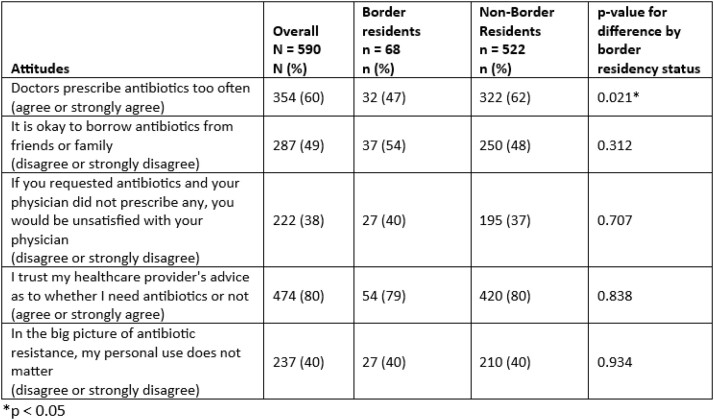
Number and percentage of survey respondants with attitudes consistent with antibiotic stewardship among United States (US) residents, stratified by residency within 100 kilometers of the US-Mexico border.

**Conclusion:**

KAP findings from this survey suggest that knowledge about antibiotic use is low regardless of proximity to the US-Mexico border. Further, practices related to antibiotic and chloroquine or hydroxychloroquine seeking in the context of COVID-19 suggest a role for public-facing information campaigns regarding medication seeking during public health emergencies.

**Disclosures:**

**All Authors**: No reported disclosures

